# What drives health mindset and expectations in the United States?

**DOI:** 10.1057/s41271-022-00382-6

**Published:** 2022-12-16

**Authors:** Laurie T. Martin, Katherine Carman, Douglas Yeung

**Affiliations:** 1 RAND Corporation, 1200 South Hayes Street, Arlington, VA 22202 USA; 2 RAND Corporation, Santa Monica, CA USA

**Keywords:** Health beliefs, Health policy, Public health

## Abstract

Health mindset is a group of beliefs or assumptions that individuals hold about the causes of health and well-being. Strengthening our understanding of factors that shape mindset and how mindset shapes expectations for who can and should be responsible for health can inform the success and sustainability of solutions to current health crises including the COVID-19 pandemic, ongoing disparities in health outcomes, and gun violence. We first examined associations between personal characteristics and experiences with health mindset. Next, we examined the association between mindset and the belief that government involvement can help address pressing health questions, using obesity as an example of a health outcome that is shaped both by personal choices and factors outside one’s control. Going forward, it will be important to consider health mindset in broader transformations of the health system and population approaches to improving health.

## Key messages

- When asked what is more important for health and well-being, over 70% of the study sample favored the role of personal choices over external factors.
- Those who believe health is largely influenced by personal choices are significantly less likely to endorse a role for government in improving health and well-being.
- While government can effectively play a role in public health and implementation of effective health policy, health mindset is a formidable barrier to the implementation of such policies.

## Introduction

With few exceptions, the United States (U.S.) is not taking advantage of the potential for collective action that could generate transformative policies, ways of thinking about budgets and funding streams, or partnerships that hold promise for widespread and impactful improvement in health. Researchers have identified five conditions of collective impact: a common agenda, shared measurement, mutually reinforcing activities, continuous communication, and backbone support. These conditions, however, are not always sufficient for large-scale transformative change [1].

In 2014, the Robert Wood Johnson Foundation (RWJF) introduced a new framework to advance a Culture of Health [2, 3]. It draws attention to the interconnected nature of health and social issues and how U.S. systems, structures, and culture shape and reinforce policies and practices that impact health and well-being. Culture of Health connotes a strong focus on the need to make health a shared value to advance and accelerate improvements in health outcomes [4]. A key driver of making health a shared value is health mindset.

Health mindset is a group of beliefs or assumptions that individuals hold about the causes of health and well-being [5, 6]. Several important factors contribute to health mindset, including an understanding of factors that generate health, such as social determinants of health; perceptions of the relative roles of personal and environmental health influences; and thoughts about equity [4]. Individual mindsets can contribute to a larger community narrative about health and ultimately influence the health priorities of a community. The mindset of individuals, particularly if that mindset is prevalent within a community, can also affect the success and sustainability of potential solutions to current and emerging health crises in the U.S. such as the COVID-19 pandemic, disparities in health, and gun violence. This is evidenced by strong reactions for and against policies to curb the COVID-19 pandemic, extend Medicaid coverage to ensure more Americans have health insurance, and require waiting periods for the purchase of guns. An assessment of whether the proposed solution aligns with one’s mindset, for example, can shape whether people embrace the solution, resist, or ignore it [9, 10]. Research shows that people are motivated to perceive the strength and credibility of solutions and messages in accordance with their predisposing beliefs and values [11, 12]. Gaining a better understanding of the relationships between mindset and the resulting expectations for who is responsible for health can help stakeholders actively working to address health challenges shape potential solutions and implementation strategies.

We examine the personal characteristics and lived experiences that shape two common health-related mindsets in the United States: (1) poor health is driven by poor choices and (2) poor health is driven by factors outside of one’s control. While the two exist and interact to produce health, this forced dichotomy is helpful for understanding these prevailing mindsets and the factors that contribute to them. Next, we explore how health mindset shapes expectations of who can and should be responsible for addressing health challenges, using obesity as an example of a health outcome well-known to be influenced by both personal choices (diet and exercise) and environmental context and conditions outside of one’s control (availability and affordability of healthy foods, safe neighborhoods).

### Poor health is driven by poor choices

Economic individualism characterizes success as stemming from hard work and self-reliance. This core value of personal responsibility, when extended to conceptualizations of health, lends itself to the parallel conclusion that individuals, rather than government, should be responsible for ensuring the health of individuals [11]. This mindset incorporates a view that changing the choices that individuals make about health behaviors, engagement with the health system, and how they spend their time, among other health-related choices, will result in improved health and well-being.

This mindset is supported by a formidable amount of evidence linking individual behaviors to health outcomes and the effectiveness of behavioral interventions to prevent disease, improve disease management, increase quality of life, and reduce health care costs [13, 14]. Such findings have resulted in powerful summary statements citing behavior as “central to the development, prevention, treatment, and management of the preventable manifestations of diseases and health conditions.” [13]. Underlying notions of individualism and personal responsibility, central to political thought in the U.S., also support this mindset [11, 15–17]. These also play roles in current health policy debates related to COVID-19, abortion, and gun control.

### Poor health is driven by factors outside of one’s control

This mindset captures core constructs of social determinants and health equity and acknowledges that while individual choices related to one’s health do impact health and well-being, not everyone is afforded the same choices; as a result, factors outside of one’s control ultimately drive poor health. Literature on health equity [18–21] posits that the “choices” that people make around their health are not available for many marginalized or disadvantaged populations due to policy, structural, or system level barriers that may have existed for generations [18].

Various models point to the importance of factors outside of one’s control, including Dahlgreen and Whitehead’s societal model [22]. It calls attention to the importance of social and community networks, living and working conditions, and general socio-economic, cultural, and environmental conditions as important for health, in addition to individual and lifestyle factors. Related to this mindset, although not the focus of this paper, are other factors that individuals believe influence health. These include fatalistic beliefs that health is “beyond one’s control and instead dependent on change, luck, fate or God.” [23].

### Does mindset shape expectations around the government’s role in improving health?

Attitudes about obesity intertwine with attitudes about personal responsibility and choices. Although the recognition of the role of environmental factors (food availability, neighborhood opportunities for exercise) is growing, many people continue to believe that obesity is the result of poor choices [16] and lack of self-discipline [24]. Some evidence suggests that this mindset—poor health is driven by poor choices—influences support for obesity policy. Associating obesity with individual choice, for example, is negatively associated with support for government policies to fight obesity [25]. Brownell and colleagues [26] argue that personal responsibility beliefs about obesity constitute “a leading basis for inadequate government efforts,” given that public health interventions may be perceived as forcing people to behave in certain ways, and thus threaten individuals’ autonomy. We saw this play out in the pandemic response, in protests against masking and vaccine mandates.

## Methods

### Data

This paper uses data from the 2018 National Survey of Health Attitudes, which RAND and RWJF developed to help understand national perspectives on health-related attitudes, values, and mindset [27, 28]. We included questions about the role of personal choices in shaping health and whether government can address obesity. We recruited respondents from two panels: the RAND American Life Panel (ALP) [29] and the KnowledgePanel (administered by Ipsos) [30]. Both panels are nationally representative Internet panels that recruit members via probability-based sampling methods. We implemented the survey identically in the two panels and the RAND Human Subjects Protection Committee approved it.

We collected data from 11 July through 30 August 2018. The combination of the two surveys resulted in a total sample of 7187 completed surveys: 2479 from the ALP and 4708 from the KnowledgePanel. We use data from a randomly selected subset of respondents (3414) who answered all questions included in our analysis. Table 1 provides an overview of the study sample used for our analyses. We conducted sensitivity analyses to investigate whether study findings were similar across the two panels (available upon request). The results were not different, supporting the decision to use a combined sample for these analyses.

**Table 1:** Summary statistics of demographic characteristics and personal experiences

Characteristic	Total sample Percent	Health mindset^a^	
		Poor choices	External factors
Age			
Under 50	36.8	35.3	40.5
50 or Over	63.2	64.7	59.5
Gender			
Male	36.0	49.4	37.7
Female	54.0	50.6	62.3
Region			
East	18.0	17.0	20.2
Midwest	22.1	21.8	22.7
South	34.7	35.7	32.4
West	25.2	25.4	24.7
Urbanicity			
Rural	13.9	14.5	12.3
Urban	86.1	85.5	87.7
Education			
Less than high school	6.0	4.9	8.6
High school	21.5	21.3	22.0
Some college including Associate's degrees	31.0	32.3	17.9
Bachelor's degree or higher	41.5	41.5	41.5
Family income			
Less than $50,000	34.8	32.2	41.2
$50,000–$99,999	32.5	32.3	33.1
$100,000+	32.7	35.6	25.7
Work status			
Working	57.4	58.4	54.9
Retired	25.8	26.1	25.1
Not working	16.8	15.5	20.0
Race/ethnicity			
Non-Hispanic White	71.4	74.3	64.9
Non-Hispanic Black	9.3	7.1	14.6
Hispanic	12.7	11.9	14.6
Non-Hispanic Asian, Pacific Islander	3.3	3.0	2.5
Non-Hispanic other	3.3	3.2	3.4
Health Insurance			
Yes	94.1	94.5	92.9
No	5.9	5.5	7.1
Self-rated health			
Excellent	9.7	10.1	8.5
Very good	39.3	41.9	33.0
Good	35.4	34.7	37.3
Fair	12.7	11.0	16.6
Poor	2.9	2.2	4.6
Impacted by poor health of other[s]			
No	58.8	60.7	54.4
Yes	41.2	39.3	45.6
Community had stressful event			
No	73.6	74.2	72.2
Yes	26.4	25.8	27.8
Discriminated against due to health			
No	87.1	89.8	80.6
Yes	12.9	10.2	19.4

### Measures

To operationalize health mindset, we asked respondents this question: “For the pair of statements below, indicate whether the FIRST statement or the SECOND statement comes closer to your own views—even if neither is exactly right.” About 71% of respondents selected “the biggest reason people in America become unhealthy is because they make poor choices that affect their health.” The remaining 29% selected “the biggest reasons people in America become unhealthy is because things outside of their control affect their health.”

Study respondents also provided information on demographic characteristics and their personal experiences with health. These included a standard self-rated health question measured on a 5-point scale from excellent to poor, and three RAND-developed questions about whether the poor health of another person affected the respondent’s life on an ongoing basis for any extended period of time; whether the respondent lived in an area that had been impacted by a significant and stressful event that might negatively impact the overall health of the people in their community; and whether the respondent had personally experienced discrimination or been treated unfairly because of an ongoing health issue, condition, or disability.

We asked participants to assess their expectations about the role government can and should play in dealing with key health issues. We selected a key issue focused on obesity for this analysis because it is impacted by both individual choices and factors that are outside the control of individuals. Specifically, we asked participants to respond to this question: “Recent research shows that as of 2018, more than one-third of American adults are obese. Which of the following levels of government do you think could do the most [e.g., through policies, programs, laws and regulations] to help reduce the number of American adults who are obese?” Respondents selected among local governments (15.3%), state governments (13.6%), federal governments (17.1%), and no government can lower this number (54.0%). For our analyses, we combined all levels of government into one category (government can help) as results were quantitatively similar when we separately considered each level of government. For all analyses, we conducted logit regressions, and present the odds ratios estimated for each coefficient.

## Results

Table 1 provides the distribution of demographic characteristics and lived experiences by health mindset. In Table 2, we present the results of our regression predicting the odds of having a health mindset that emphasizes poor choices, rather than factors outside of one’s control, as the biggest reason people become unhealthy.

**Table 2:** Logit regression predicting health mindset as a function of beliefs, characteristics, and lived experiences

Factor	Health mindset: people are unhealthy because of poor choices OR [95% CI]
Age	
Under 50	Ref.
50 or over [relative to under 50]	1.22 [1.02–1.47]**
Gender	
Male	Ref.
Female	0.64 [0.55–0.75]***
Race/ethnicity	
Non-Hispanic White	Ref.
Non-Hispanic Black	0.46 [0.36–0.59]***
Hispanic	0.82 [0.64–1.05]
Non-Hispanic Asian or Pacific Islander	1.21 [0.75–1.95]
Non-Hispanic other	0.97 [0.63–1.49]
Region	
East	Ref.
Midwest	1.12 [0.88–1.42]
South	1.43 [1.14–1.78]***
West	1.27 [1.00–1.61]*
Location	
Rural	Ref.
Urban	0.87 [0.69–1.10]
Education	
Less than HS	Ref.
High school	1.47 [1.04–2.07]**
Some college including Associate’s degrees	1.73 [1.23–2.44]***
Bachelor’s degree or higher	1.12 [0.79–1.59]
Family income, annual	
Less than $50,0000	Ref.
$50,000–$99,999	1.05 [0.86–1.27]
$100,000 +	1.44 [1.15–1.78]***
Work status	
Currently working	Ref.
Retired	0.89 [0.72–1.09]
Not working	1.02 [0.81–1.27]
Health insurance	
No	Ref.
Yes	1.07 [0.77–1.49]
Self-rated health	
Excellent	Ref.
Very good	1.06 [0.80–1.41]
Good	0.84 [0.63–1.12]
Fair	0.65 [0.46–0.90]**
Poor	0.57 [0.34–0.93]**
Impacted by others poor health	
No	Ref.
Yes	0.84 [0.72–0.99]**
Community had stressful event	
No	Ref.
Yes	0.97 [0.81–1.16]
Discriminated against due to health	
No	Ref.
Yes	0.61 [0.48–0.76]***
Constant	2.35 [1.33–4.14]**
Observations	3414

All regressions are logits. Coefficients are odds ratios, confidence intervals in parentheses

***p < 0.01, **p < 0.05, *p < 0.1

Having the mindset that poor health stems largely from poor choices is associated with being 50 years or older relative to those who are younger, living in the south or west relative to those living in the east, and having an annual income over $100,000 relative to those with income under $50,000 per year. The relationship between health mindset and education is U-shaped with little difference between those with the highest and lowest levels of education. Those with a high school degree or some college education were more likely than those with no high school to report that poor choices are more important to poor health than factors outside of one’s control. However, females and those who are black were significantly less likely to have this mindset compared to males and those who are white, respectively.

With respect to lived experiences, people in fair or poor health are less likely to endorse a mindset that prioritizes personal factors as key drivers of health relative to those in excellent health. Those who have been impacted by other people’s health are also less likely to endorse personal factors as the main reason people are unhealthy as are those that report being discriminated against as a result of their health. These data suggest that individuals who have experienced a health problem, either directly or indirectly, are more likely to have a health mindset that associates poor health with external factors.

Associations between health mindset and beliefs about whether the government could address obesity appear in Table 3. After controlling for demographic characteristics and personal health experiences, individuals whose health mindset prioritized poor choices as the primary reason people are unhealthy are about half as likely to believe that any level of government can help reduce obesity.

**Table 3:** Logit regression predicting expectations of governments as a function of health mindset, characteristics, and lived experiences

Factor	Governments can help address obesity OR [95% CI]
Health mindset–people are unhealthy because of	
Factors outside of one’s control	Ref.
Poor choices	0.43 [0.37–0.51]**
Age	
Under 50	Ref.
50 or Over [relative to under 50]	0.44 [0.37–0.52]**
Gender	
Male	Ref.
Female	0.98 [0.85–1.14]
Race/ethnicity	
Non-Hispanic White	Ref.
Non-Hispanic Black	1.84 [1.42–2.39]**
Hispanic	2.11 [1.66–2.68]**
Non-Hispanic Asian or Pacific Islander	1.77 [1.15–2.74]**
Non-Hispanic other	1.07 [0.71–1.61]
Region	
East	Ref.
Midwest	0.77 [0.61–0.98]*
South	0.74 [0.60–0.92]**
West	0.85 [0.67–1.06]
Location	
Rural	Ref.
Urban	1.11 [0.89–1.38]
Education	
Less than HS	Ref.
High school	0.85 [0.60–1.21]
Some college	1.38 [0.98–1.95]
Bachelor’s degree or higher	2.50 [1.76–3.55]**
Family income, annual	
Less than $50,0000	Ref.
$50,000–$99,999	0.86 [0.71–1.04]
$100,000+	0.92 [0.75–1.13]
Work status	
Currently working	Ref.
Retired	0.84 [0.69–1.02]
Not working	1.01 [0.82–1.26]
Health insurance	
No	Ref.
Yes	1.12 [0.81–1.54]
Self-rated health	
Excellent	Ref.
Very good	1.07 [0.83–1.39]
Good	1.08 [0.82–1.40]
Fair	0.77 [0.56–1.07]
Poor	0.82 [0.49–1.35]
Impacted by others poor health	
No	Ref.
Yes	1.04 [0.89–1.21]
Community had stressful event	
No	Ref.
Yes	0.99 [0.84–1.17]
Discriminated against due to health	
No	Ref.
Yes	1.13 [0.90–1.43]
Constant	1.51 [0.86–2.63]
Observations	3414

**p < 0.01, *p < 0.05

## Discussion

The U.S. is at a challenging point in time with respect to the health of the nation. Improving health and well-being will require collective action. Critical to collective action is a need to better understand how an individual’s unique experiences contribute to shaping their health mindset, and to appreciate how the health mindset of individuals and the prevailing health mindset of communities shape beliefs about, and support for, potential programmatic and policy solutions. Without this understanding, and without an appreciation of such connections, the U.S. risks continuing to implement strategies that lack strong support. This lack of support may lessen the strategy’s effectiveness, and could create further polarization around the issue.

The idea that the way people think about the causes of health influences their support of health-related policies is not new [31]. Few studies, however, have examined such associations with nationally representative data. Our study shows that those whose health mindset favors the role of personal choices over external factors—71% of our study sample—are significantly less likely to endorse a role for government. While evidence suggests that the government can effectively play a role in public health and implementation of effective health policy [32, 33], health mindset is a formidable barrier to the implementation of such policies.

Our research has several important limitations. First, this study is cross-sectional, thus we cannot interpret these results as causal. Second, we asked respondents to pick between two statements of health mindset. Some respondents may not feel that either statement fully resonates with their viewpoints and that both personal choices and factors outside of one’s control contribute to poor health. Given the importance of health mindset for collective action, more work is needed to refine our understanding of this construct and its measurement. Finally, our survey sample was drawn from two separate online panels. The survey modules, however, were identical, with the same ordering of questions, formatting on the screen, and randomizations. Sensitivity analyses suggested that there were no meaningful differences in results when data were examined separately by survey or when survey version was accounted for in the analytic model.

Going forward, it will be important to consider health mindset in the development of policies and strategies to support the health and well-being of all people living in the U.S., in specific public health interventions, and in messaging to inform our understanding of factors that influence health [34]. Such approaches may be informed by work from Brownell and colleagues [16] who note that information could be provided in a way that suggests these seemingly dichotomous health mindsets can in fact come together in “complementary, if not synergistic” approaches, where government action can set the context to “create more optimal defaults that support informed and responsible decisions and hence enhance personal freedoms.” Policy initiatives that emphasize community control over their environment such as community-wide participation in local health planning may be another strategy [35].

## Conclusion

While most Americans likely share a common objective of improved health and well-being in their communities, health mindset is an important, but much understudied, factor in shaping strategies and solutions to strengthen the health of our nation.

## Data Availability

The datasets analysed during the current study will be made available in the ICPSR repository in 2023.
